# Formation of tyrosine radicals in photosystem II under far-red illumination

**DOI:** 10.1007/s11120-017-0442-3

**Published:** 2017-09-18

**Authors:** Nigar Ahmadova, Fikret Mamedov

**Affiliations:** Molecular Biomimetics, Department of Chemistry – Ångström Laboratory, Uppsala University, Box 523, 751 20 Uppsala, Sweden

**Keywords:** Photosystem II, Tyrosine Z and D, Electron transfer, Far-red light

## Abstract

Photosystem II (PS II) contains two redox-active tyrosine residues on the donor side at symmetrical positions to the primary donor, P_680_. Tyr_Z_, part of the water-oxidizing complex, is a preferential fast electron donor while Tyr_D_ is a slow auxiliary donor to P_680_^+^. We used PS II membranes from spinach which were depleted of the water oxidation complex (Mn-depleted PS II) to study electron donation from both tyrosines by time-resolved EPR spectroscopy under visible and far-red continuous light and laser flash illumination. Our results show that under both illumination regimes, oxidation of Tyr_D_ occurs via equilibrium with Tyr_Z_^•^ at pH 4.7 and 6.3. At pH 8.5 direct Tyr_D_ oxidation by P_680_^+^ occurs in the majority of the PS II centers. Under continuous far-red light illumination these reactions were less effective but still possible. Different photochemical steps were considered to explain the far-red light-induced electron donation from tyrosines and localization of the primary electron hole (P_680_^+^) on the Chl_D1_ in Mn-depleted PS II after the far-red light-induced charge separation at room temperature is suggested.

**Electronic supplementary material:**

The online version of this article (doi:10.1007/s11120-017-0442-3) contains supplementary material, which is available to authorized users.

## Introduction

Solar energy is successfully utilized by plants, algae, and bacteria in the process called photosynthesis. In oxygenic photosynthesis, solar energy is converted to chemical energy in the form of carbohydrates and O_2_ is released as a byproduct (Kern and Renger 2007; Renger and Renger 2008; Vinyard et al. 2013). The initial reaction of photosynthesis takes place in photosystem II (PS II), a multicomponent Chl protein complex embedded in the thylakoid membrane of chloroplasts and cyanobacteria. The active PS II complex is made from 25 protein subunits and host a chain of the redox-active cofactors involved in the key water oxidation reaction and subsequent electron transfer (Umena et al. 2011; Wei et al. 2016). These cofactors are bound by the PS II central core which is composed of the D_1_ and D_2_ proteins, the inner pigment–protein antenna complexes CP43 and CP47, Cyt b_559_, and several low molecular weight essential subunits (Danielsson et al. 2006; Umena et al. 2011; Suga et al. 2015). On the luminal side, water-oxidizing complex of plants and algae is shielded by three extrinsic proteins PsbO, PsbP, and PsbQ (Bricker et al. 2012).

The sequence of electron transfer reactions leading to the oxidation of water occurs in the following order. After light absorption by antenna, P_680_ is excited and rapidly loses an electron to the nearby primary electron acceptor Pheo. Reduced Pheo^−^ passes an electron to the bound plastoquinone Q_A_, forming Q_A_^−^ which in turn transfers an electron to Q_B_ (Renger and Renger 2008). All these cofactors are single electron carriers while exchangeable plastoquinone Q_B_ can accept two electrons and then become double protonated upon reduction. Reduced Q_B_H_2_ diffuses from the Q_B_-pocket and is replaced by another plastoquinol from the membrane PQ pool (Renger and Renger 2008; Barber 2016). On the donor side of PS II, the water-oxidizing complex is composed of Mn_4_CaO_5_ cluster and redox-active tyrosine D1-161 (Tyr_Z_), and mostly bound by the D1 protein. P_680_^+^ is a strong oxidant with redox potential of 1.25 V, high enough to drive water oxidation reaction via Tyr_Z_ (Grabolle and Dau 2005; Cardona et al. 2012). Water oxidation occurs at the Mn_4_CaO_5_ cluster which goes through S-cycle to oxidize water to a molecular O_2_ and four protons by transferring four electrons to P_680_^+^ via Tyr_Z_ (Rappaport et al. 2002; Renger and Renger 2008; Vinyard et al. 2013).

In intact oxygen-evolving PS II Tyr_Z_^•^ oxidation has half-time in nsec–µsec range (Brettel et al. 1984; Renger 2012). In the absence of Mn_4_CaO_5_ the half-time became by 2–3 orders of magnitude slower (Babcock and Sauer 1975a; Brettel et al. 1984). PS II contains another redox-active tyrosine D2-160 (Tyr_D_), which is located symmetrically to Tyr_Z_ on the D2 protein (Styring et al. 2012). Contrary to Tyr_Z_^•^, Tyr_D_^•^ is very stable and stays oxidized in the dark for minutes to hours (Babcock and Sauer 1973; Styring and Rutherford 1987; Vass and Styring 1991). Due to the slow oxidation under physiological pH, Tyr_D_ is not competitive to Tyr_Z_ as an electron donor to P680^+^. However, at elevated pH with a pK_a_ ~ 7.6, Tyr_D_ becomes a very efficient donor, with half-times comparable to those seen for Tyr_Z_, (*t*_1/2_ = 190 ns) (Faller et al. 2001, 2002). The difference in the environment of two tyrosines is the reason for their difference in oxidation kinetics (Umena et al. 2011). Tyr_D_ is in relative hydrophobic environment, deeply buried in the protein interior. On the contrary, Tyr_Z_ is in more hydrophilic surrounding with a cluster of water molecules nearby (Ferreira et al. 2004; Umena et al. 2011; Suga et al. 2015). Interestingly, Tyr_D_ has only a single water molecule nearby which can take two positions (2.6–3.1 and 4.3–4.5 Å) (Umena et al. 2011; Saito et al. 2013; Suga et al. 2015; Sjöholm et al. 2016; Ahmadova et al. 2017).

**Scheme 1:**
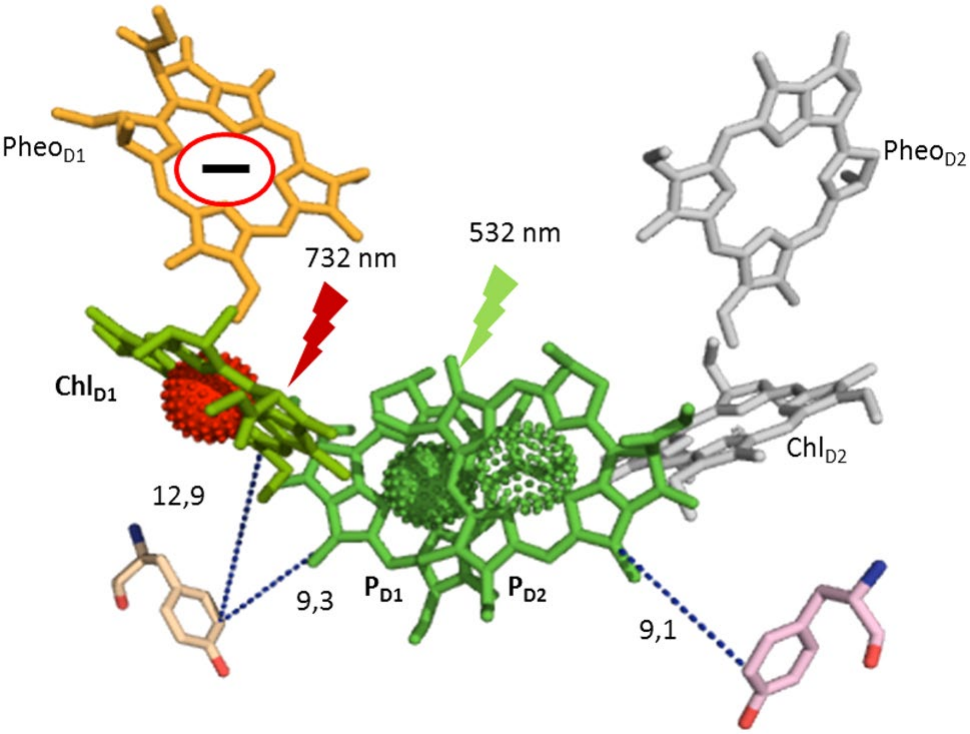
Electron transfer components and primary charge separation events in PS II under visible and far-red light illumination. Numbers indicate distances between components in Å

The primary donor in PS II, P_680_ consists of four Chl molecules bound by the D1/D2 heterodimer denoted as P_D1_, P_D2_, Chl_D1_, and Chl_D2_, (see Scheme 1). The distance from reaction center Chl P_D1_ and P_D2_ to the Tyr_Z_ and Tyr_D_, respectively is 9.1–9.3 Å. Both tyrosines can be oxidized by P_680_ entity after light-triggered primary charge separation formed P_680_^+^ Pheo^−^ pair. It is still under debate which Chl in P_680_ entity forms the primary donor (Rappaport and Diner 2008). Interestingly, apart from visible-light-driven charge separation, the far-red light-driven charge separation up to 800 nm was reported in PS II (Thapper et al. 2009). Taking into account the lower energy of the far-red photons an alternative primary charge separation event was proposed (Thapper et al. 2009). It was also shown that at low temperature the primary charge pair formed under the far-red light illumination is Chl_D1_^+^ Pheo^−^ (Hughes et al. 2006; Romero et al. 2012; Mokvist et al. 2014; Novoderezhkin et al. 2016). This conclusion was made based on the different yields of the electron donor pathways in PS II at 5 K (Mokvist et al. 2014). The situation at physiological temperatures is unclear (Reimers et al. 2016).

The slow oxidation behavior of Tyr_D_ makes it possible to study by the conventional EPR spectroscopy while Tyr_Z_ oxidation kinetics is too fast to be followed. Different biochemical treatment such as Tris and NH_2_OH washing can remove the Mn_4_CaO_5_ cluster and extrinsic proteins, leaving PS II core exposed to the lumen (Boussac and Etienne 1982b; Gadjieva et al. 1999; Mamedov et al. 2007). This makes Tyr_Z_ oxidation to slow down by two or three orders of magnitude (t_1/2_ = 20–600 ms) (Buser et al. 1990). In this case both tyrosine donors can be accessed by EPR spectroscopy. Symmetrically situated at the different sides of P_680_, they constitute a useful, simplified system to study the far-red-induced photochemistry at room temperature (Scheme 1).

In the present work we investigate the primary charge separation through oxidation of two tyrosines under two different excitation wavelengths. We have studied tyrosine oxidation kinetics in the Mn-depleted PS II membranes at different pH values. The difference in the oxidation efficiency of Tyr_Z_ and Tyr_D_ allowed us to suggest localization of the primary electron donor Chl in P_680_ after far-red illumination at physiological temperature.

## Materials and methods

### Sample preparation

PS II-enriched membranes (BBY type) were isolated from hydroponically grown spinach (*Spinacia oleracea*) by the method of Berthold et al. 1981 with some modifications according to Völker et al. (1985). The samples were resuspended in a 25 mM MES buffer, pH 6.1, 400 mM sucrose, 15 mM NaCl, and 3 mM MgCl_2_ at a Chl concentration of 5–6 mg/mL and stored at −80 °C until use. All sample preparations were performed in darkness or under the dim green light.

**Fig. 1:**
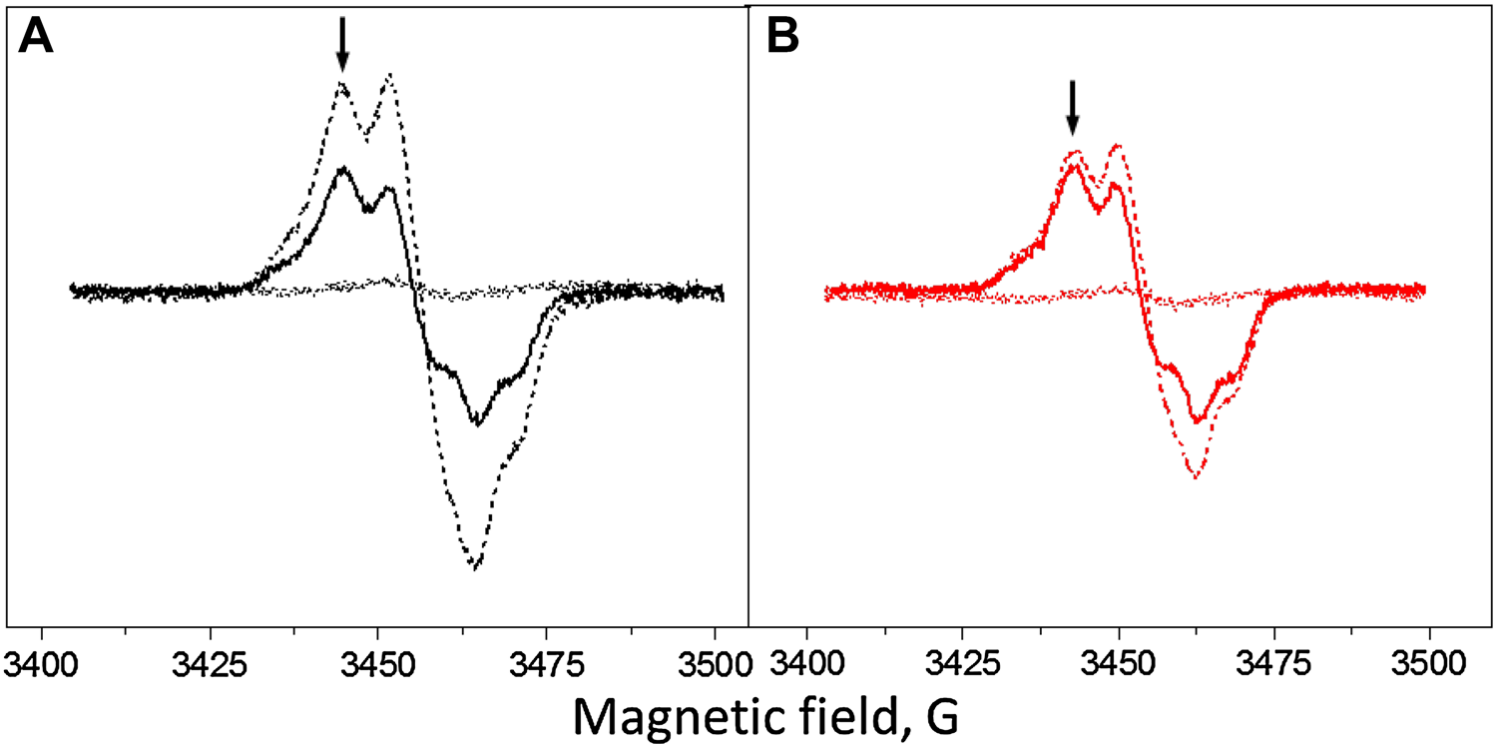
EPR spectra of the Tyr radicals from the Tris-washed PS II membranes at pH 8.5 induced by white light illumination (**A**, black spectra) or far-red light illumination (**B**, red spectra). Spectra shown are after 10 h dark incubation at room temperature (*dotted line spectra*), during continuous illumination (*dashed line spectra*) and after 5 min of dark incubation (*solid line spectra*). The *arrows* indicate the field position (3465 G) for the kinetics measurements. EPR conditions: microwave frequency 9.75 GHz, microwave power 8 mW, modulation amplitude 5 G, temperature 293 K

The Mn_4_CaO_5_ cluster and extrinsic subunits were removed by the Tris washing (Gadjieva et al. 1999, Mamedov et al. 2007). PS II membranes were resuspended in 1.0 M Tris buffer at pH 9.1 with Chl concentration 1 mg/mL. They were stirred at 4 °C for 30 min under room light. After centrifugation, the pellet was washed twice with a low molar buffer containing 2 mM MES–NaOH pH 6.1, 300 mM sucrose, 10 mM NaCl, 3 mM MgCl_2_, and stored at −80 °C. This treatment washed away >90% of bound Mn and all three extrinsic proteins (Gadjieva et al. 1999). Tyr_D_ reduction in Tris-washed PS II samples was obtained by incubation samples at a concentration of 4–5 mg of Chl/mL in the dark for 10 h at room temperature (21 °C). The incubation treatment reduced about 95% of the Tyr_D_ (Fig. 1).

For the experiments samples were diluted to 2 mg Chl/mL by addition of the appropriate amount of high molar measuring buffer, containing 300 mM sucrose, 10 mM NaCl, and 3 mM MgCl_2_ with 150 mM of either glutamic acid (pH 4.7), MES (pH 6.3), or glycylglycine (pH 8.5). The final buffer concentration after addition of high molar buffer was 25 mM.

Steady-state oxygen evolution activity was measured with a Hansatech Clark-type electrode at 20 µg of Chl/mL in a measuring buffer at pH 6.1. 2 mM potassium ferricyanide K_3_[Fe(CN)_6_] and 0.5 mM PpBQ were used as electron acceptors. The activities of the PSII membrane preparation was ∼550 µmol of O_2_× (mg of Chl)^−1^ × h^−1^ (pH 6.3), while Tris-washed samples did not show any oxygen evolution.

### EPR measurements

Room temperature EPR measurements were performed with ELEXSYS E500 spectrometer (Bruker Biospin GmbH) equipped with a SuperX bridge and a super high Q SHQE4122 cavity. The measurements were done in a 250 μL quartz flat cell at a sample concentration of ∼2 mg of Chl/mL. Steady-state Tyr_Z/D_ oxidation was monitored after induction with LED setup (white or far-red light, see below) mounted at the EPR cavity window at field position of 3465 G. In addition, Tyr_Z/D_ oxidation kinetics was triggered with a 6 ns, 100 mW, 523 or 732 nm laser flash given to the sample. Data analysis was performed with the Bruker Xepr 2.1 software. The standard error in the signal amplitude estimation in our EPR measurements was less than 5%.

### Light sources

Samples were illuminated with two kinds of LED: white and far-red light. White light LED had one major emission peak at 450 nm and two smaller peaks at 549 and 600 nm. Far-red light LED had an emission peak at 732 nm. Two cut-off Schott filters CC4 and RG9 were used with far-red LED to ensure no visible-light contamination (see Supplementary Fig. 1S for the actual output spectra of used LED light sources). LEDs were set up at the EPR cavity window. 532 and 732 nm laser flashes (20 mJ, pulse duration 6 ns, pulse bandwidth ±0.1 nm) were provided by the Quanta-Ray MOPO-730 optical parametric oscillator, driven by Nd: YAG laser (Spectra Physics, USA).

## Results

Tyr_D_^•^ is much less stable in the Mn-depleted PS II than in the active PS II samples with the full O_2_-evolving activity, where reduction of Tyr_D_^•^ requires application of the reducing agents (Vass and Styring 1991; Sjöholm et al. 2016; Ahmadova et al. 2017). The dark incubation of our Tris-washed PS II membranes for 10 h at room temperature resulted in the reduction of 90–95% of Tyr_D_^•^ (Fig. 1, dotted line spectra). Under illumination, both tyrosines can be observed by the conventional EPR spectroscopy; however, their different kinetic properties allow to clearly distinguish between Tyr_D_^•^ and Tyr_Z_^•^ after light is switched off. Moreover, similar EPR properties of both radicals in the absence of the Mn cluster allow to quantify Tyr_Z_^•^ on the basis of Tyr_D_^•^ radical (Boussac and Etienne 1982a, 1984; Roffey et al. 1994).

Illumination of the sample for 2 min with white light at pH 8.5 and subsequent 5 min dark adaptation resulted in full oxidation of Tyr_D_^•^ (Fig. 1A, solid line spectrum). Any induced Tyr_Z_^•^ has decayed during 5 min of dark adaptation (decay half-time of Tyr_Z_^•^ is ca 600 ms in Tris-washed PS II at this pH, Table 1) and any decay of Tyr_D_^•^ is negligible. Thus, the resulted spectrum is taken for 100% of Tyr_D_^•^ and was used in further quantifications. When measurements were performed under illumination conditions, tyrosine radical spectrum arose from Tyr_D_^•^ (100%) and the additional intensity, which is attributed to the Tyr_Z_^•^ radical (75%, Fig. 1A, dashed line spectrum). When measurements were done under far-red illumination, the additional intensity from Tyr_Z_^•^ amounted to only 15% (Fig. 1B, dashed line spectrum).

**Table 1:** Fitted half-times and amplitudes of Tyr_Z_^•^ exponential decay after induction by 532 and 732 nm laser flash in the Tris-washed PSII membranes without any additions or in the presence of 2 mM ferricyanide (Ferri) and 2 mM ferricyanide and 1 mM DPC (Ferri + DPC)

t_1/2_, ms (Ampl., %)	pH 4.7			pH 6.3			pH 8.5		
	No add.	Ferri	Ferri + DPC	No add.	Ferri	Ferri + DPC	No add.	Ferri	Ferri + DPC
**532 nm**	22 ± 2 ms (34%)	22 ± 2 ms (85%)	10 ± 1.5 ms (42%)	222 ± 25 ms (43%)	118 ± 15 ms (95%)	17 ± 2 ms (55%)	557 ± 53 ms (48%)	436 ± 49 ms (109%)	59 ± 7 ms (25%)
**732 nm**	21 ± 2 ms (18%)	24 ± 2 ms (20%)	6 ± 1 ms (8%)	170 ± 21 ms (19%)	102 ± 13 ms (27%)	24 ± 2 ms (25%)	596 ± 53 ms (20%)	338 ± 37 ms (37%)	76 ± 7 ms (16%)

The standard error in the signal amplitude estimation in our EPR measurements was <5%

**Fig. 2:**
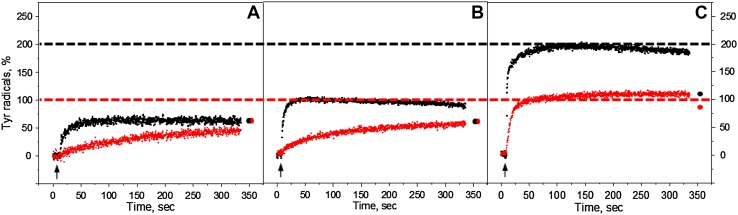
Kinetics of tyrosine oxidation recorded at 3465 G under continuous white light (*black traces*) or far-red illumination (*red traces*) in the reduced Mn-depleted PS II membranes at pH 4.7 (**A**), pH 6.3 (**B**), and pH 8.5 (**C**). Each trace represents an average of four single measurements in independent samples. The amplitude of tyrosine radical after 5 min dark incubation is indicated as a *black* or *red circle* at the end of the each trace. The *arrows* indicate starting time point of illumination. EPR conditions: microwave frequency 9.75 GHz, microwave power 8 mW, modulation amplitude 5 G, temperature 293 K

The kinetics of Tyr_D_ and Tyr_Z_ oxidation were measured by monitoring the EPR signal induction at 3465 G (arrow in Fig. 1) under continuous illumination at three different pH values (Fig. 2). The field position chosen for kinetic measurement is lying outside of the magnetic field range in which signals from Chl and Car cations could contribute (Visser et al. 1977; Hanley et al. 1999).

### Tyrosine oxidation under continuous white or far-red light illumination

Steady-state oxidation of Tyr_D_ and Tyr_Z_ residues in the Mn-depleted PS II membranes under continuous illumination with white or far-red light are shown in Fig. 2. The oxidation kinetics was accelerated towards the higher pH values under both illumination conditions. The total yield of tyrosine radical formation was also pH dependent and increased towards high pH. The maximum formation of tyrosine radicals was observed at pH 8.5 (Fig. 2C).

Under white light illumination, the steady-state level of tyrosine oxidation reached maximum at any given pH quite fast within ca 50 s (Fig. 2, black traces). The maximum corresponded to 65% at pH 4.7, 100% at pH 6.3, and 195% at pH 8.5. Almost 200% of oxidation indicates that full induction of both Tyr_Z_^•^ and Tyr_D_^•^ was achieved at high pH. Some decay of the steady-state level of the signal was observed during illumination after 50 s at pH 6.3 (~5%) and 100 s at pH 8.5 (~10%) (Fig. 2B, C, black traces). This decay is due to the full reduction of the acceptor side which leads to the backflow of electrons from Q_A_^−^ or Q_B_^−^ to Tyr_Z_^•^ and/or photoinhibition. Such decay was not observed in the presence of exogenous acceptor (see below). Interestingly, after the light was turned off and 5 min of dark incubation of white light illuminated samples, no decay of tyrosine amplitude was observed at pH 4.7 (Fig. 2A, black dot). While at higher pH, the amplitude of tyrosine signal decreased to 67% at pH 6.3 and to 105% at pH 8.5 (Fig. 2B, C, black dots).

The kinetics of tyrosine oxidation under the far-red illumination was different from the kinetics under the white light illumination at all three pH values investigated. Oxidation of tyrosine was much slower at pH 4.7 and 6.3 (Fig. 2A, B, red traces). However, the sharp rise has been observed at pH 8.5 and was comparable to the white light oxidation rise (Fig. 2C, red trace). The total yield of Tyr^•^ under far-red light illumination was 47% at pH 4.7, 60% at pH 6.3, and 105% at pH 8.5. At pH 4.7 and 6.3 Tyr oxidation never reached the steady-state level even after illumination for 325 s (Fig. 2A, B, red traces), whereas at pH 8.5, the oxidation reached half of the level of tyrosine oxidation under white light illumination very fast within 50 s and was continued to slowly rise afterwards (Fig. 2C, red trace). Incubation of far-red illuminated samples for 5 min resulted in the additional rise of tyrosine amplitude at pH 4.7 and 6.3 to 67% (Fig. 2A and B, red dot). In contrast, at pH 8.5 decrease of tyrosine amplitude to 91% was observed (Fig. 2C, red dot).

To conclude this part, continuous illumination of the reduced Mn-depleted PSII membranes with white light resulted in the formation of stable Tyr_D_^•^ radical at pH 4.7 and 6.3 as could be judged from its post-illumination stability. The formation was much slower and less effective under far-red light illumination if compared with white light illumination. In contrast, at pH 8.5 illumination with white light resulted in the formation of both Tyr_Z_^•^ radical and Tyr_D_^•^ radical, while illumination with far-red light resulted in albeit fast but only Tyr_D_^•^ formation. Thus, the only conditions which resulted in the observation of semi-stable Tyr_Z_^•^ were high pH and white light.

### Flash-induced tyrosine signal formation

**Fig. 3:**
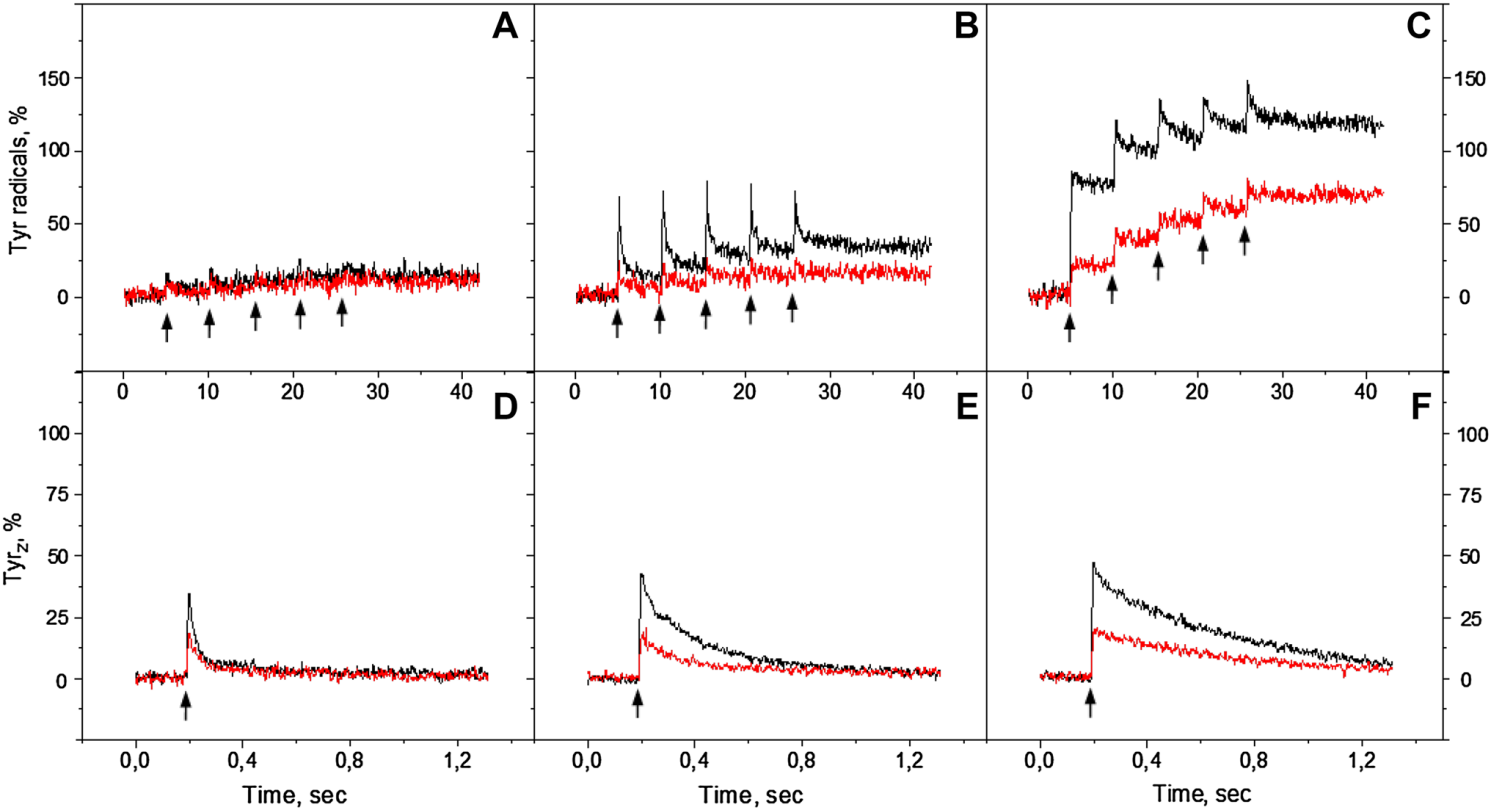
Tyr_Z_ and Tyr_D_ oxidation kinetics at 3465 G in the reduced Mn-depleted PS II membranes, induced by a train of five 532 nm (*black traces*) or 732 nm laser flashes (*red traces*) at pH 4.7 (**A**), pH 6.3 (**B**) and pH 8.5 (**C**). Each trace represents an average of four single measurements in independent samples. Tyr_Z_ oxidation kinetics induced by single 532 nm (*black traces*) or 732 nm laser flash (*red traces*) at: pH 4.7 (**D**), pH 6.3 (**E**) and pH 8.5 (**F**). Each trace represents an average of 199 flashes. EPR conditions are the same as in Fig. 2

In order to further investigate the sequence of tyrosine radical formation in PS II, the monochromatic laser flashes at 532 and 732 nm were used. Figure 3A-C show the kinetic of Tyr_Z_^•^ and Tyr_D_^•^ formation and decay after five consecutive flashes separated by 5 s interval (indicated by arrows). Tyr^•^ was formed after each flash is divided into the decaying and non-decaying tyrosine signal. According to the decay half-times of Tyr_Z_^•^ and Tyr_D_^•^ in the Mn-depleted PSII preparations, the decaying part of tyrosine signal is attributed to Tyr_Z_^•^ and non-decaying part to Tyr_D_^•^ (Babcock and Sauer 1975a; Vass and Styring 1991). At pH 4.7, with green and far-red flashes, Tyr_Z_ and Tyr_D_ oxidation took place in 5–10% of PS II centers (Fig. 3A). Similarly, very small induction of Tyr_Z_^•^ and Tyr_D_^•^ was observed at pH 6.3 with far-red flashes (15–20%, Fig. 3B, red trace). With green flashes at pH 6.3 however, both tyrosines were induced in the substantial number of PS II centers, resulting in 41% of non-decaying Tyr_D_^•^ (black trace). At pH 8.5 significant amount of both tyrosines was formed at both wavelengths as could be judged from the decaying and non-decaying parts similarly to what was observed under continuous illumination. Again the final amplitude of Tyr_D_^•^ induced by green flashes (117%) was higher than induced by far-red flashes (72%, Fig. 3C).

Contribution of Tyr_Z_^•^ to the total oxidation process was studied in more detail in the same samples after Tyr_D_ oxidation was completed (Fig. 3D-F). After accumulation, it is clear that Tyr_Z_^•^ signal was inducible at all three pHs by both 532 and 732 nm wavelengths. The amplitude of Tyr_Z_^•^ was twice higher after green flashes if compared to far-red flashes. Similarly to the continuous illumination experiments, Tyr_Z_ oxidation was pH dependent and was small at pH 4.7 and increased towards pH 8.5. This difference was pronounced with green flash induction (Fig. 3D-F, black traces). Tyr_Z_^•^ signal decay was also pH dependent and slowed down towards higher pHs with both wavelengths, from 21 ms at pH 4.7 to 596 ms at pH 8.5 (Fig. 3D-F; Table 1).

### Influence of exogenous electron acceptor

**Fig. 4:**
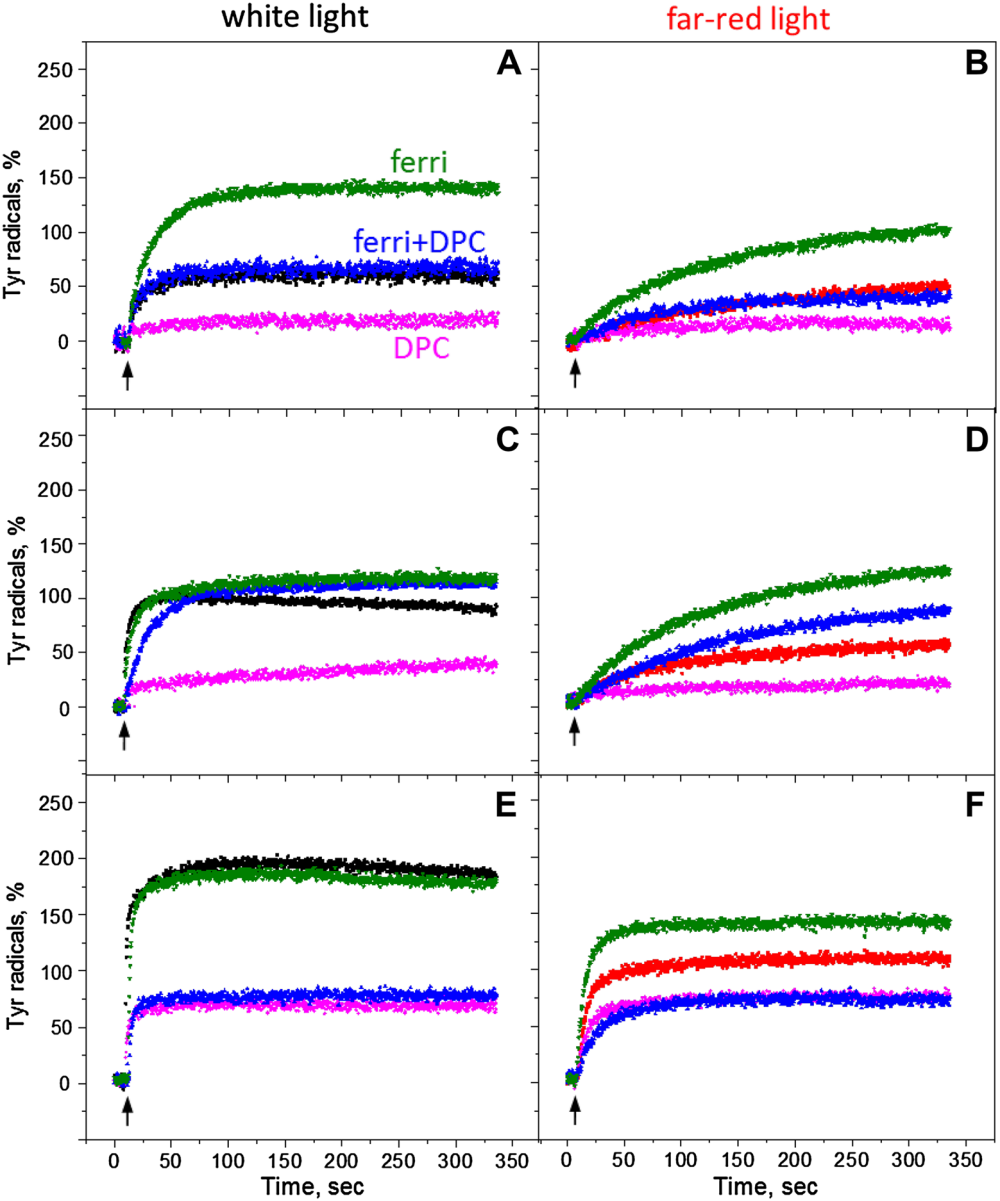
Kinetics of tyrosine oxidation recorded under white light (**A, C, E**, *black traces*) or far-red light illumination (**B, D, F**, *red traces*) at pH 4.7 (**A, B**), pH 6.3 (**C, D**) and pH 8.5 (**E, F**). *Pink traces*—in the presence of 1 mM DPC; *green traces*—in the presence of 2 mM ferricyanide and *blue trace*—in the presence of 1 mM DPC and 2 mM ferricyanide. EPR conditions are the same as in Fig. 2

Better oxidation of tyrosines could be achieved by addition of ferricyanide which also prevents the loss of charge separation by recombination of Q_A_^−^ Tyr_Z_^•^ and Q_B_^−^ Tyr_Z_^•^ states (Bishop and Spikes 1955; Delrieu and Rosengard 1989). We have measured oxidation of tyrosine in the presence of ferricyanide in order to further understand the tyrosine oxidation in Mn-depleted PS II under continuous illumination with white and far-red light. Figure 4 (green traces) show the oxidation kinetics in the presence of ferricyanide at pH 4.7, 6.3 and 8.5 respectively. At pH 4.7 the amplitude of tyrosine oxidation was twice higher in the presence of ferricyanide if compared to a trace without any addition (Fig. 4, green traces). However, under far-red illumination, the kinetics was slower, but more efficient, and still rising after 325 s of illumination (Fig. 4B, green trace).

The kinetics of tyrosine oxidation measured at pH 6.3 with the ferricyanide addition under white light was very similar to the kinetics obtained without any additions (100% oxidation), except for the absence of the small decay after the maximum amplitude was reached (Fig. 4C, green and black traces). This is reasonable since the presence of the acceptor is preventing decay to the steady-state equilibrium between tyrosine oxidation and recombination reactions. Under far-red light however, the oxidation kinetics were much slower and rose to the higher level in the presence of ferricyanide than without any additions and reached the same amplitude (100%) as under white light illumination (Fig. 4D, green trace). At higher pH, under white light illumination, the oxidation kinetics and amplitude were again very similar in the presence or absence of ferricyanide reaching ca 200% indicating full oxidation of both Tyr_Z_ and Tyr_D_ (Fig. 4E, green and black traces). Interestingly, under far-red light at pH 8.5, the amplitude was higher than without acceptor almost reaching 142% (Fig. 4F, green trace) if compared to the black trace without additions (100%). This indicates that in addition to the full induction of Tyr_D_^•^, 50% of Tyr_Z_^•^ was induced by far-red light at pH 8.5 in the Mn-depleted PS II centers. The rise of the signal was also fast and comparable to the white light induction at these conditions.

### Influence of exogenous electron donor

**Scheme 2:**
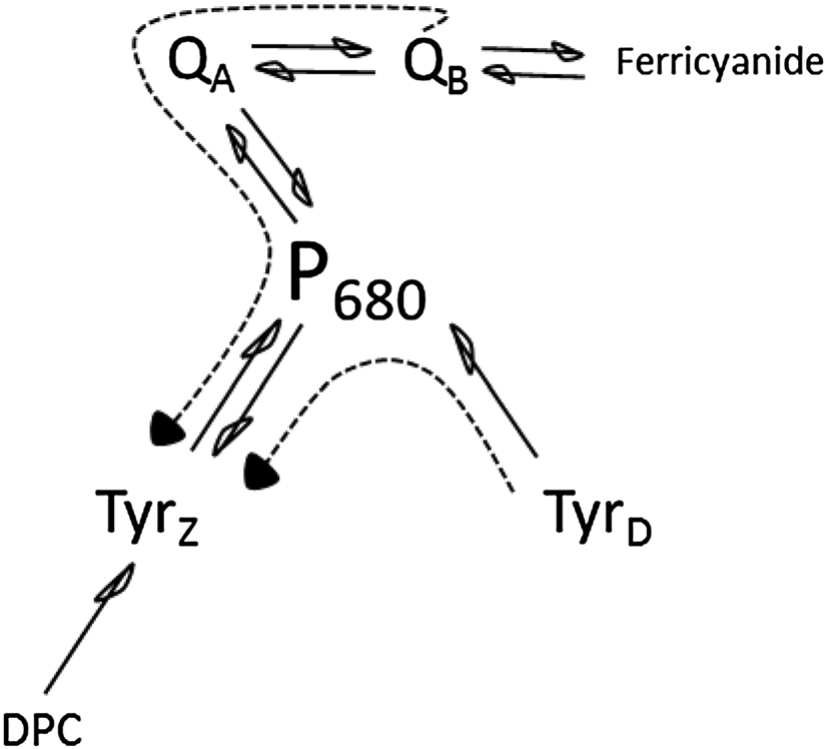
Electron transfer events and redox equilibria leading to Tyr_D_ and Tyr_Z_ oxidation in the Mn-depleted PS II centers

To distinguish between direct (by P_680_^+^) or indirect (via equilibrium with Tyr_Z_^•^, see Scheme 2) oxidation of Tyr_D_, DPC as an exogenous electron donor to Tyr_Z_^•^ was added to the samples before illumination. It is known that addition of DPC accelerates the Tyr_Z_^•^ lifetime and this makes it less available for Tyr_D_ oxidation (Babcock and Sauer 1975b; Yerkes and Babcock 1980; Roffey et al. 1994). Figure 4A-D (pink traces) show oxidation kinetics under continuous white and far-red light illumination in the presence of DPC at pH 4.7 and 6.3, respectively. It is clear that addition of DPC significantly inhibited tyrosine oxidation in the majority of PS II centers under both illuminating conditions. At pH 6.3 oxidation was slightly better than at pH 4.7; however, at both pHs tyrosine oxidation did not reach the complete equilibrium and the kinetics were still rising after 325 s of both white and far-red light illumination (Fig. 4A-D, pink traces). Interestingly, at pH 8.5 the tyrosine induction was better and both white and far-red light illumination have the same effect on oxidation which reached 70% of the PS II centers (Fig. 4E, F, pink traces).

We also performed these measurements in the presence of both exogenous donor and acceptor, DPC and ferricyanide, to see how the steady-state equilibrium between tyrosine oxidation and recombination reaction will be affected (Fig. 4, blue traces). The effect was very pH dependent. At pH 4.7 the presence of both DPC and ferricyanide resulted in higher tyrosine amplitude than in the presence of only DPC under both light conditions (Fig. 4A, B, blue traces). It restored amplitude to the level which was achieved in the measurements without any additions (compare to black and red traces). At pH 6.3 the effect was bigger and final amplitude of Tyr^•^ was significantly higher than in the presence of only DPC and 25–35% higher than in a sample without any additions (Fig. 4, compare black, pink, and blue traces (C) and red, pink, and blue traces (D). The oxidation kinetics under white light was however slowed down (Fig. 4C, blue trace). At pH 8.5 addition of ferricyanide had no effect and the final oxidation level was very similar to the level obtained only in the presence of DPC (Fig. 4E, F, pink and blue traces).

It should be noted that only Tyr_D_^•^ was formed in the presence of DPC or DPC and ferricyanide under both white and far-red light as can be estimated from the residual signal after the light was switched off (not shown). We were not able to observe any fast decaying Tyr_Z_^•^ and the signal was never higher than 100% at all pH values measured. Our conclusion from these measurements is that DPC is an effective donor to Tyr_Z_^•^ at all three pH values and the addition of ferricyanide only eliminates the recombination reaction from the acceptor side of PS II which takes place at low pH values (Ahmadova et al. 2017).

### Flash-induced tyrosine signal formation in the presence of donor and acceptor

**Fig. 5:**
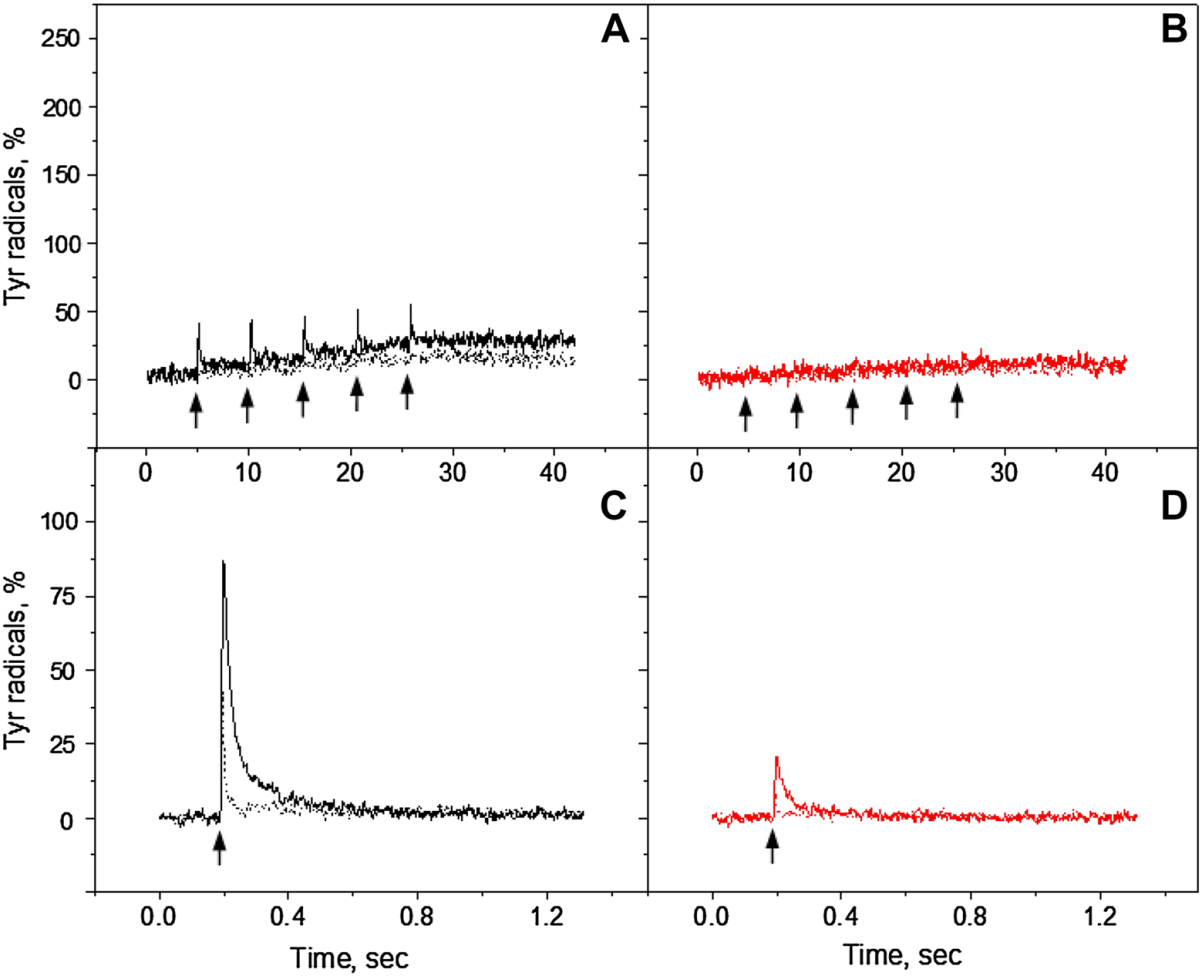
Tyr_Z_ and Tyr_D_ oxidation kinetics in the reduced Mn-depleted PS II membranes, induced by a train of five 532 nm (**A**, black traces) or 732 nm laser flashes (**B**, red traces) at pH 4.7 in the presence of 2 mM ferricyanide (solid line) or in the presence of 2 mM ferricyanide and 2 mM DPC (dotted line). Tyr_Z_ oxidation kinetics induced by single 532 nm (**C**, black traces) or 732 nm laser flash (**D**, red traces) at pH 4.7 in the presence of 2 mM ferricyanide (solid line) or in the presence of 2 mM ferricyanide and 2 mM DPC (dotted line). EPR conditions are the same as in Fig. 3

**Fig. 6:**
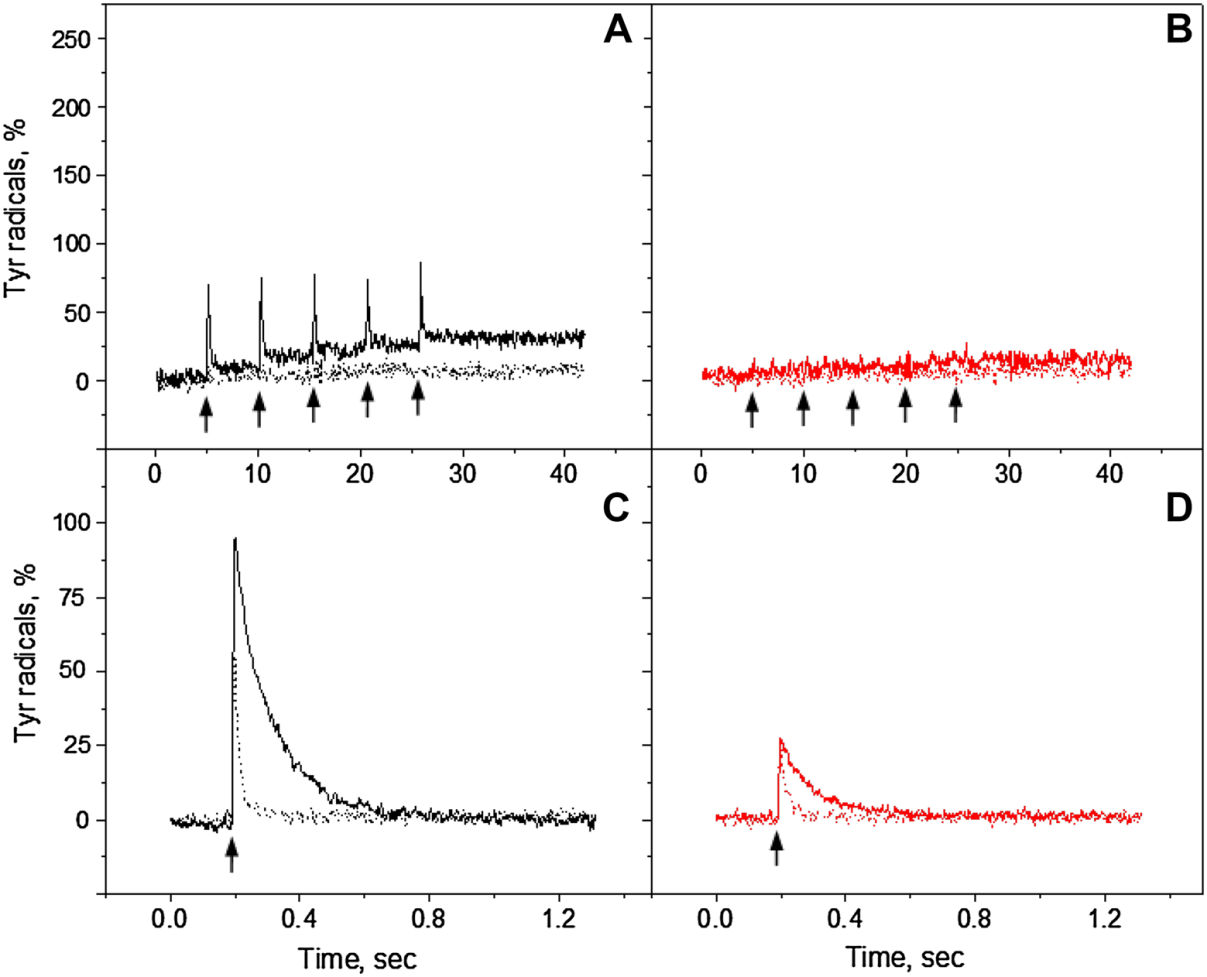
Tyr_Z_ and Tyr_D_ oxidation kinetics in the reduced Mn-depleted PS II membranes, induced by a train of five 532 nm (**A**, black traces) or 732 nm laser flashes (**B**, red traces) at pH 6.3 in the presence of 2 mM ferricyanide (solid line) or in the presence of 2 mM ferricyanide and 2 mM DPC (dotted line). Tyr_Z_ oxidation kinetics induced by single 532 nm (**C**, black traces) or 732 nm laser flash (**D**, red traces) at pH 6.3 in the presence of 2 mM ferricyanide (solid line) or in the presence of 2 mM ferricyanide and 2 mM DPC (dotted line). EPR conditions are the same as in Fig. 3

**Fig. 7:**
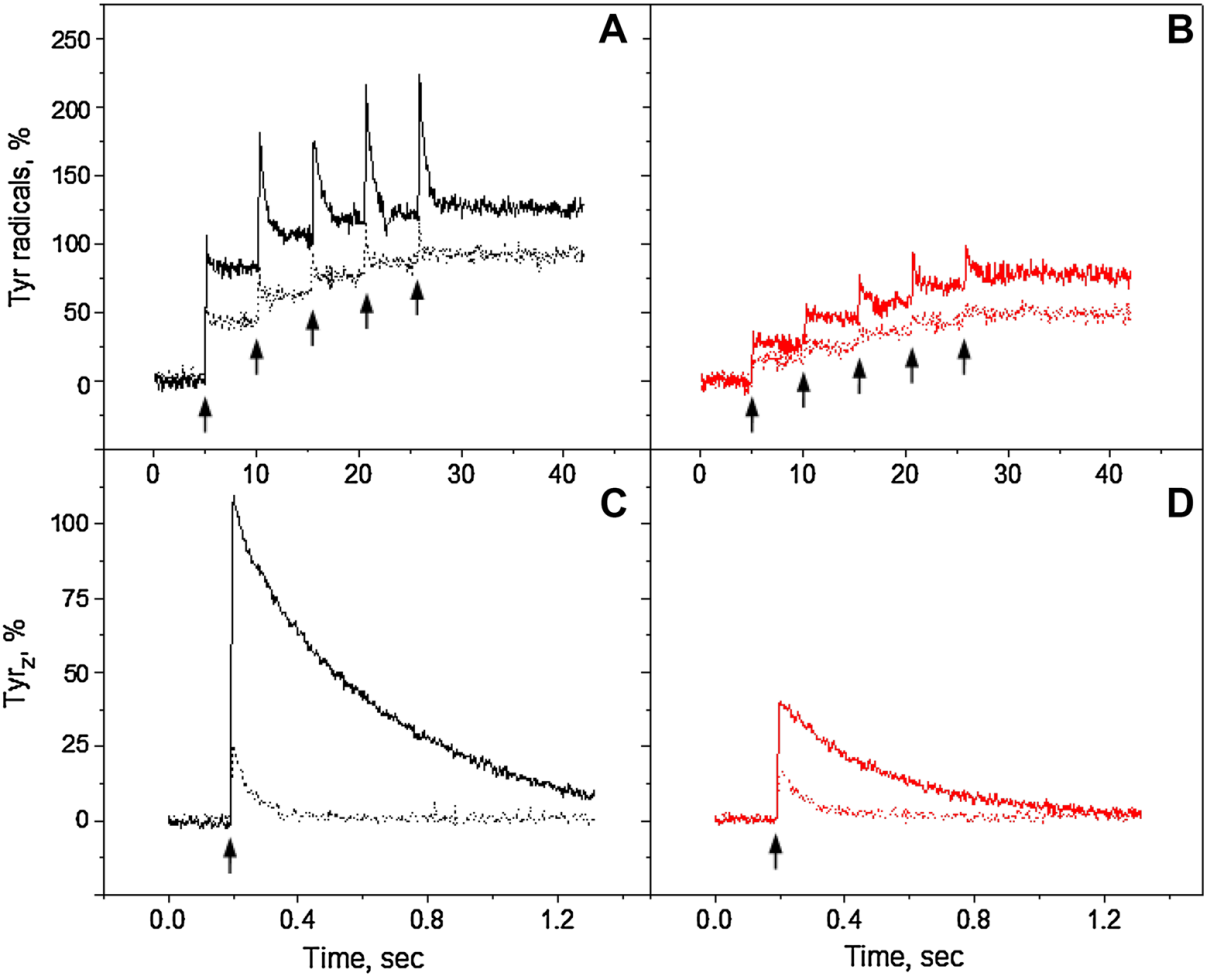
Tyr_Z_ and Tyr_D_ oxidation kinetics in the reduced Mn-depleted PS II membranes, induced by a train of five 532 nm (**A**, black traces) or 732 nm laser flashes (**B**, red traces) at pH 8.5 in the presence of 2 mM ferricyanide (solid line) or in the presence of 2 mM ferricyanide and 2 mM DPC (dotted line). Tyr_Z_ oxidation kinetics induced by single 532 nm (**C**, black traces) or 732 nm laser flash (**D**, red traces) at pH 8.5 in the presence of 2 mM ferricyanide (solid line) or in the presence of 2 mM ferricyanide and 2 mM DPC (dotted line). EPR conditions are the same as in Fig. 3

Figures 5, 6, and 7 show the kinetics of Tyr_Z_^·^ and Tyr_D_^·^ formation and decay after five consecutive laser flashes separated by 5 s each (indicated by arrows) in the presence of electron donor and acceptor. In the presence of only ferricyanide we observed Tyr_Z_ oxidation at pH 4.7 and 6.3 from the first given green flash (Figs. 5A, 6A, black solid traces). At pH 8.5 oxidation was very efficient and resulted in complete oxidation of Tyr_Z_ with consequent decay and of Tyr_D_ (Fig. 7A, black solid trace). Interestingly, no fast decay was observed after the first flash, similar to measurements without any additions (Fig. 3C, black trace).

Interestingly, we did not detect Tyr_Z_ oxidation with single far-red flashes in the presence of ferricyanide and final Tyr_D_ oxidation was less than 12% at pH 4.7 or at pH 6.3 (Figs. 5B, 6B, red solid traces). At pH 8.5 with far-red flashes, we observed almost full oxidation of Tyr_D_ after five flashes (80%, Fig. 7B, red solid trace). Unlike in oxidation with green flashes, the fast decay kinetics (Tyr_Z_^•^) was observed on the third flash and onward but seemingly the first two flashes induced only Tyr_D_ oxidation in the majority PS II centers at high pH (48%, Fig. 7B, red solid trace).

Accumulated Tyr_Z_^•^ signal induced by green or far-red flashes in the presence of ferricyanide is shown in Figs. 5, 6, and 7C and D, solid traces. With 532 nm induction, the amplitude of Tyr_Z_^•^ slightly increased towards high pH and corresponded to 85% at pH 4.7, 95% at pH 6.3, and 109% at pH 8.5. With 732 nm induction, we observed only 20% at pH 4.7, 27% at pH 6.3, and 37% at pH 8.5. The decay half-times were also pH dependant and corresponded to 22 ms at pH 4.7, 118 ms at pH 6.3, and 436 ms at pH 8.5, Figs. 5, 6, and 7C and D, solid traces, Table 1. The decay half-time was independent of the induction wavelength.

We also investigated the kinetics of flash-induced tyrosine signal formation in the presence of both DPC and ferricyanide (Figs. 5, 6, 7A and B, dotted traces). The addition of DPC almost completely inhibited the tyrosine formation at pH 4.7 and 6.3. We did not observe any Tyr_Z_^•^ formation in the presence of DPC and the final Tyr_D_^•^ formation was less than 5% at these two pH values (Figs. 5, 6A and B, dotted traces). At pH 8.5, the Tyr_Z_^•^ signal was still unresolvable in the presence of DPC but Tyr_D_ oxidation occurred in the majority of the PS II centers with 532 nm flashes (>75%, Fig. 7A, black-dotted trace) and in less centers with 732 nm flashes (45%, Fig. 7B, red-dotted trace). Figures 5, 6, and 7C and D show that in the presence of DPC the Tyr_Z_^•^ induction is strongly inhibited and very short lived (ca 20 ms or less, Table 1) at low pH values, while at high pH the half-time was less affected and significant loss of Tyr_Z_^•^ amplitude could be attributed to competitive reduction of P_680_^+^ by Tyr_D_ (Scheme 2) (Fig. 7C, D, dotted traces).

## Discussion

The special Chl molecules, P_680_, serve as a primary electron donor in PS II. P_680_ is a tetrameric pigment entity which comprises four Chl molecules. The central Chl pair, P_D1_ and P_D2_ are weakly excitonically coupled and situated at 30° angle to the horizontal plane. The other two Chls, Chl_D1_ and Chl_D2_ are occupying symmetrical positions at 10 Å center-to-center distance each from P_D1_ and P_D2_, respectively (Scheme 1) (Umena et al. 2011; Suga et al. 2015; Wei et al. 2016). The localization of excitation energy in P_680_ and the first Chl^+^ electron donor formed after the “standard” charge separation conditions (visible-light excitation) have been extensively studied by different spectroscopic methods (Zech et al. 1997; Diner et al. 2001; Groot et al. 2005; Holzwarth et al. 2006; Romero et al. 2010, 2012). The primary hole is consensually placed on the P_D1_ Chl (P_D1_^+^) (Scheme 1) (Hillmann et al. 1995; Diner et al. 2001; Schlodder et al. 2008b; Cardona et al. 2012) although some groups reported that weak spectral differentiation among all four Chls might lead to a distribution of the excitation energy at ambient temperature (Romero et al. 2010, 2012). There are also reports that the reduction of Pheo_D1_, which is the primary acceptor in PS II (Scheme 1) occurs prior to the oxidation of P_D1_/P_D2_ (Groot et al. 2005; Holzwarth et al. 2006).

The far-red photochemistry at low temperature has been suggested to induce different primary charge pair, Chl_D1_^+^ Pheo^−^ (Mokvist et al. 2014). This was based on the different donation efficiency to P680^+^ from Tyr_Z_ and Cyt b_559_/Chl_Z_ pathways under green and far-red illumination at 5 K, in the so-called product analysis of the charge-separated state (Mokvist et al. 2014). It seems that nature of the primary electron hole in P_680_^+^ varies depending on the temperature and excitation wavelength (Raszewski et al. 2008). In this paper, we investigate if the similar effect of excitation wavelength (visible vs. far-red light) on the primary donor occurs at physiological conditions. Tyr_Z_ and Tyr_D_, which are symmetrically positioned at about 9.2 Å distance from the central P_D1_ and P_D2_ Chls, respectively, (Scheme 1) were used as competing electron donors to elucidate the nature of the primary charge-separated state at room temperature.

The task is complicated by the fact that there are two electron transfer pathways for Tyr_D_ to be oxidized in the Mn-depleted PS II. The first one is occurring via Tyr_Z_^•^ which is formed rapidly after oxidation by P_680_^+^. Tyr_D_, with its lower redox potential, is then slowly oxidized in the following reaction (Boussac and Etienne 1982c, 1984; Faller et al. 2001):

1 $$Tyr_{D} Tyr_{Z} \xrightarrow{\text{light}}Tyr_{D} Tyr_{Z}^{\bullet} \to Tyr_{D}^{\bullet} Tyr_{Z}$$

This oxidation pathway occurs at low and middle pH values. However, at high pH (above pK_a_ of Tyr_D_ (Vass 1991; Faller et al. 2001; Ahmadova et al. 2017)), direct Tyr_D_ oxidation by P_680_^+^ was reported in the Mn-depleted preparation in at least half of the PS II centers (Faller et al. 2001). As a result, partial localization of the electron hole on P_D2_ Chl was suggested (Faller et al. 2001).

Our data show that under continuous illumination we mostly observed oxidation of Tyr_D_ (except white light illumination at high pH where both Tyr_Z_^•^ and Tyr_D_^•^ were formed (Fig. 2)). The pH dependence of Tyr_D_^•^ induction was similar to what was reported on green flash-induced Tyr_D_^•^ formation in intact PS II (Vass and Styring 1991; Sjöholm et al. 2016; Ahmadova et al. 2017). In our Tris-washed PSII membranes, under continuous white light illumination Tyr_D_ oxidation takes place in 65 and 100% at pH 4.7 and 6.3, respectively (Fig. 2A, B, black traces). With far-red light illumination (732 nm LED light), oxidation at these pH values was much slower and less effective (Fig. 2A, B, red traces). The diminished formation of Tyr_D_ under far-red light could be due to either less effective charge separation or due to the different nature of the primary donor which makes the far-red photochemistry less effective.

The first hypothesis seems unlikely because under 732 nm light most of the PS II centers undergo charge separation. As it was shown by (Thapper et al. 2009) illumination with 730 nm light resulted in P_680_^+^ Pheo^−^ primary charge pair formation in the vast majority of the PS II centers. This is by far in more centers than the decreased tyrosine radical formation under similar 732 nm illumination (Fig. 2A, B). Thus, the possibility of the second hypothesis cannot be ruled out. At pH 8.5 continuous white light illumination induces both tyrosines. Interestingly, illumination with 732 nm light resulted in only Tyr_D_^•^ formation (Fig. 2C, red trace). Full induction of Tyr_D_^•^ (100%) also indicates that far-red light excites 100% of the PS II centers, as mentioned above. The difference between tyrosine formation at pH 8.5 under visible and far-red illumination could originate either from different tyrosine oxidation pathways (Tyr_D_ vs. Tyr_Z_) or different localizations of the primary donor. Here our data with the addition of exogenous donor and acceptor to regulate the “redox pressure” in and out from Tyr_Z_^·^ (Scheme 2) will help to answer these questions.

The addition of ferricyanide increased oxidation amplitude of Tyr_D_ at low pH by efficiently preventing recombination from the acceptor side of PS II (Fig. 4, green traces). At pH 6.3 and 8.5, the effect of ferricyanide addition was only observable under the far-red illumination (Fig. 4D, F). Since we also observed that the addition of ferricyanide also increased the amplitude of Tyr_Z_^•^ induction (Table 1), it is clear that final oxidation of Tyr_D_, at pH 4.7 and 6.3, takes place via Tyr_Z_^•^ as described in reaction (1). More importantly, this indicates that recombination reaction was more efficiently prevented by the ferricyanide addition under far-red light illumination. This implies different recombination partners formed under the far-red light on the donor side of PS II. Since Tyr_Z_^•^ is the same at both illumination conditions, the only difference could be assumed at the P_680_^+^ entity.

The most important and informative results were obtained when measurements were done in the presence of DPC. DPC is known to be an efficient electron donor to Tyr_Z_^•^ in the Mn-depleted PS II preparations (Babcock and Sauer 1975b; Yerkes and Babcock 1980; Roffey et al. 1994). In our case, both Tyr_D_ and DPC are competing for the Tyr_Z_^•^ reduction and in the presence of DPC the decay half-time of Tyr_Z_^•^ is significantly decreased (Table 1); thus, effectively blocking indirect Tyr_D_ oxidation (Scheme 2). The addition of DPC severely inhibited Tyr_D_^•^ formation at pH 4.7 and 6.3 under both white and far-red illuminations (Fig. 4A-D, pink traces). DPC inhibition of the Tyr_D_^•^ formation was more effective under far-red light illumination (only 18 and 19% formation at pH 4.7 and 6.3, respectively). Interestingly, even under white light illumination at pH 8.5 only Tyr_D_ oxidation was observed in the presence of DPC and no extra intensity could be attributed to the Tyr_Z_ oxidation (Fig. 4E, F, pink traces). This is another indication for the direct oxidation of Tyr_D_ by P_680_^+^ at high pH (Scheme 2) (Faller et al. 2001).

In the presence of both DPC and ferricyanide, the oxidation kinetics was found to be similar to what was found in the absence of any additions (Fig. 4A-D, blue traces). In this case, availability of the electron donor (DPC) and acceptor (ferricyanide) to and from Tyr_Z_^•^ allowed the same final steady-state oxidation level, thus, effectively restoring the original “redox pressure” on Tyr_Z_^•^.

Flash-induced Tyr_Z_^•^ oxidation was observed at all pHs and under both 532 and 732 nm laser flash in our Tris-washed PS II membranes. It was both pH dependent (as was reported before (Boska et al. 1983)) and wavelength dependent (Table 1; Figs. 3E, D, and 5, 6, 7C and D). The decay half-time of Tyr_Z_^•^ was pH dependent (Babcock and Sauer 1975a; Shigemori et al. 1997) but wavelength independent (Table 1). This indicates that after the Tyr_Z_^•^ formation (if any), its consequent reduction either from Tyr_D_^•^ or DPC (when present) or by recombination from the acceptor side (Scheme 2) was independent on the way of how the primary charge separation occurred. At pH 4.7 and 6.3 the presence of DPC decreased the amplitude of Tyr_Z_^•^, especially under 732 nm flash (to final 3% and 25%, respectively if compared to 532 nm flash (Figs. 5, 6C and D; Table 1)). This resulted in significantly less Tyr_D_^•^ formation, especially after far-red flashes (Figs. 5, 6A and B). In contrast at high pH 8.5, even if the effect of DPC addition on Tyr_Z_^•^ kinetics was similarly dramatic, a significant amount of Tyr_D_^•^ was formed. The absence of the fast decay on the first flash in Fig. 7A, B points out to the direct oxidation of Tyr_D_^•^ by P_680_^+^ under both green and far-red (although much less efficiently) flashes.

Thus, our data indicate that different photochemistry is involved in oxidation of two tyrosines in the Mn-depleted PS II membranes. The far-red light-induced photochemistry is taking place in the majority of the PS II centers. At normal pH values, it results in the decrease of Tyr_Z_^•^ formation and correspondingly higher recombination rate under the far-red light illumination. Recombination reaction takes place between the acceptor side of PS II (Q_A_^−^ or Q_B_^−^) and P_680_^+^ which prevents Tyr_Z_^•^ formation. The reason for this could be a different primary charge separation event and correspondingly the localization of the electron hole in P_680_^+^ (Scheme 1).

The central Chl pair, P_D1_ and P_D2_ is excitonically weakly coupled. The greater physical separation, the slight differences in tetrapyrrole ring orientation, and the smaller dipole strength of the Q_y_ transition cause weaker electronic interaction between special Chl pair (Diner and Rappaport 2002; Raszewski et al. 2005; Schlodder et al. 2008a). This is why the Chl pair, P_D1_ and P_D2_ do not represent the lowest energy sink for the excitation energy (Diner and Rappaport 2002; Raszewski et al. 2005; Schlodder et al. 2008a). Whereas the monomeric Chl_D1_ has the lowest site energy because of the absence of such coupling (Schlodder et al. 2008a). The far-red light bears low excitation energy if compared to white light. Therefore, far-red light-induced excitation migration among four Chls in P_680_ would be an energetically less favorable process. It is more likely that under the far-red light the excitation localized on Chl_D1_, closer to the primary electron acceptor, Pheo (Scheme 1) was shown to take place at very low temperatures (Mokvist et al. 2014).

Localization of the electron hole on Chl_D1_ is not an ideal situation since recombination from the acceptor side Chl_D1_^+^ Q_A_^−^ is faster and efficiently quenches productive charge separation. On the other hand, Tyr_Z_ is still in the close distance to both Chl_D1_ and P_D1_ to have an efficient donation in the centers where Chl_D1_^+^ is still available. Moreover, at physiological temperatures, as soon as Chl_D1_ loses an electron, the hole can migrate to the neighboring P_D1_ and P_D2_. After the hole jumps on P_D1_, the localization of the hole on P_D1_ or P_D2_ becomes a very random process. This why at high pH direct oxidation of Tyr_D_ by P_680_^+^ (via P_D2_^+^ as was suggested by Faller et al. 2001) is possible at least in part of the PS II centers under the far-red light illumination.

Thus, our results indicate that in the Mn-depleted PS II at room temperature the primary charge separation pathway under the far-red excitation occurs via Chl_D1_^+^ Phe_D1_^−^ primary pair, similar to what was reported for the active PS II at ultra-low temperature (Mokvist et al. 2014). The question if the same reaction occurs in the active PS II under physiological conditions requires further investigations.

## Electronic supplementary material

Below is the link to the electronic supplementary material.

Supplementary material 1 (DOCX 178 KB)

## Abbreviations

Chl: Chlorophyll
Car: Carotenoid
DPC: Diphenylcarbazide
EPR: Electron paramagnetic resonance
Ferri: Potassium ferricyanide K_3_[Fe(CN)_6_]
PS II: Photosystem II
Pheo: Pheophytin
Q_A_ and Q_B_: Primary and secondary plastoquinone acceptor in PSII
Tyr_Z_: Tyrosine 161 on the D1 protein
Tris: Tris(hydroxymethyl)aminomethane
Tyr_D_: Tyrosine 160 on the D2 protein
