# Supplementary material for: Formation of tyrosine radicals in photosystem II under far-red illumination
Source: Photosynth Res. 2017 Sep 18;136(1):93–106. doi: 10.1007/s11120-017-0442-3 (PMC5851703; doi:10.1007/s11120-017-0442-3)
Supplement: Supplementary file 1 — Supplementary material 1 (DOCX 178 KB) [file 11120_2017_442_MOESM1_ESM.docx]

**Supplementary figure 1SA.** White light LED actual output spectra used in our study.


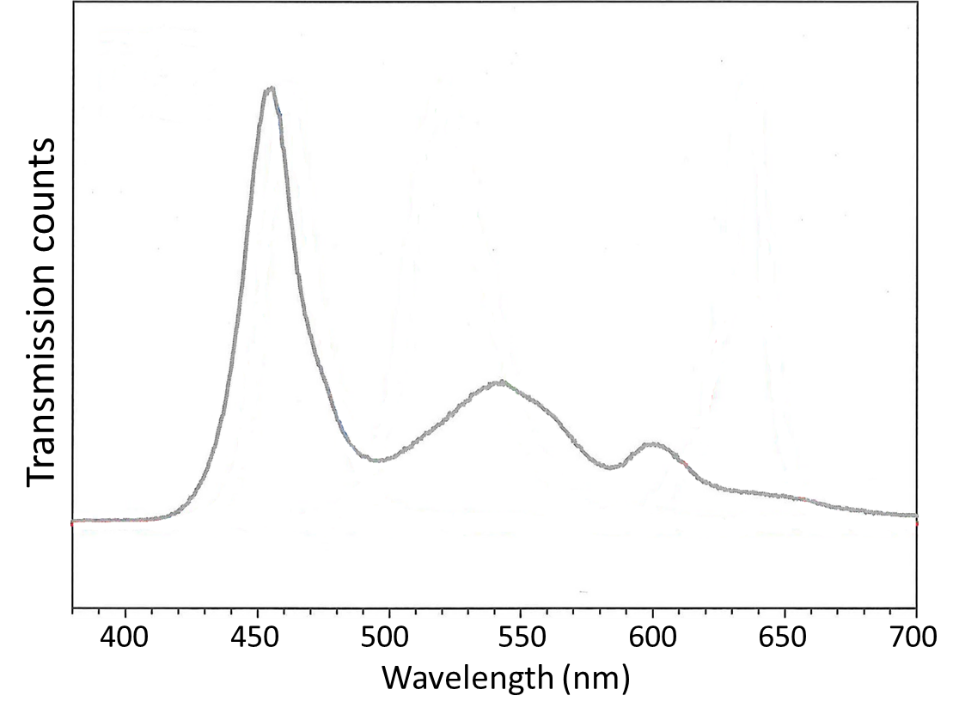

**Supplementary figure 1SB.** Far-red light LED actual output spectra without any filters (red line) and with two cut off Schott filters CC4 and RG9 (black line) used in our study.
